# APC/C-Cdh1-targeted substrates as potential therapies for Alzheimer’s disease

**DOI:** 10.3389/fphar.2022.1086540

**Published:** 2022-12-14

**Authors:** Rebeca Lapresa, Jesus Agulla, Juan P. Bolaños, Angeles Almeida

**Affiliations:** ^1^ Institute of Functional Biology and Genomics, CSIC, University of Salamanca, Salamanca, Spain; ^2^ Institute of Biomedical Research of Salamanca, University Hospital of Salamanca, CSIC, University of Salamanca, Salamanca, Spain

**Keywords:** Cdh1, neurodegeneration, Alzheimer’s disease, molecular targets, therapy

## Abstract

Alzheimer’s disease (AD) is the most prevalent neurodegenerative disorder and the main cause of dementia in the elderly. The disease has a high impact on individuals and their families and represents a growing public health and socio-economic burden. Despite this, there is no effective treatment options to cure or modify the disease progression, highlighting the need to identify new therapeutic targets. Synapse dysfunction and loss are early pathological features of Alzheimer’s disease, correlate with cognitive decline and proceed with neuronal death. In the last years, the E3 ubiquitin ligase anaphase promoting complex/cyclosome (APC/C) has emerged as a key regulator of synaptic plasticity and neuronal survival. To this end, the ligase binds Cdh1, its main activator in the brain. However, inactivation of the anaphase promoting complex/cyclosome-Cdh1 complex triggers dendrite disruption, synapse loss and neurodegeneration, leading to memory and learning impairment. Interestingly, oligomerized amyloid-β (Aβ) peptide, which is involved in Alzheimer’s disease onset and progression, induces Cdh1 phosphorylation leading to anaphase promoting complex/cyclosome-Cdh1 complex disassembly and inactivation. This causes the aberrant accumulation of several anaphase promoting complex/cyclosome-Cdh1 targets in the damaged areas of Alzheimer’s disease brains, including Rock2 and Cyclin B1. Here we review the function of anaphase promoting complex/cyclosome-Cdh1 dysregulation in the pathogenesis of Alzheimer’s disease, paying particular attention in the neurotoxicity induced by its molecular targets. Understanding the role of anaphase promoting complex/cyclosome-Cdh1-targeted substrates in Alzheimer’s disease may be useful in the development of new effective disease-modifying treatments for this neurological disorder.

## Introduction

Alzheimer Disease’s (AD) is a progressive neurodegenerative disorder and the leading cause of dementia in the adult, affecting around 35 million patients. The number of people with AD is growing rapidly and it is estimated that the number of patients with dementia will double in Europe and triple worldwide, by 2050 (Scheltens et al., 2021). Increasing prevalence coupled with a lack of effective treatments make this disease an important socio-economic problem for states and families (Cummings 2021).

The irreversible brain damage is the result of neurodegeneration in selective brain areas, primarily hippocampus and cortex, underlying the gradual decline of cognitive function and daily living activities (Mrdjen et al., 2019). The two major histopathological hallmarks of AD are protein aggregates of amyloid-beta (Aβ) peptide forming extracellular senile plaques and intracellular neurofibrillary tangles (NFT) of hyperphosphorylated tau protein (Scheltens et al., 2021). Although senile plaques are not correlated with cognitive decline, Aβ oligomers formed during the peptide aggregation are synaptotoxic and trigger disease progression (Ferreira et al., 2015). The accumulation of these oligomers starts years before AD clinical symptoms (Panza et al., 2019). In this context, synaptic plasticity impairment is an early pathological event in AD and strongly correlates with the cognitive status of the patient. Moreover, synaptic dysfunction and loss usually arises in prodromal or mild cognitive impairment (MCI) stages of the AD continuum. This synaptic loss is followed by neurodegeneration and cognitive decline progression in later stages of the disease (Jackson et al., 2019; Karisetty et al., 2020). Aβ aggregates trigger synaptic dysfunction mainly through excitotoxicity *via* different mechanisms, including Ca^2+^ influx into the neurons, internalization, and removal of glutamate receptors (GluRs), mitochondrial dysfunction, energy deficit, and oxidative/nitrosative stress, all leading to synaptic plasticity disruption and neurodegeneration. Long-term potentiation (LTP) and long-term depression (LTD), which are the two primary forms of synaptic plasticity, are altered in AD (Zhang et al., 2022).

The ubiquitin-proteasome system (UPS) controls protein homeostasis, which is essential for key cellular proccesses, including cell cycle, cell survival, metabolism, inflammation, and synaptic plasticity, among others (Dantuma and Bott 2014; Cheng et al., 2018). The role of the UPS on synaptic plasticity has been confirmed by the fact that pharmacologic or genetic inhibition of the UPS causes defects in the LTP and LTP-related behaviour (Jiang et al., 1998; Fonseca et al., 2006). Impaired UPS has been linked with both the early and late stages of AD. Specifically, Cdh1, the main activator of the E3 ubiquitin ligase Anaphase Promoting Complex/Cyclosome (APC/C) in neurons, is a key regulator of synaptic plasticity and neuronal survival (Almeida 2012; Huang and Bonni 2016; Bobo-Jimenez et al., 2017). Cdh1 deficiency triggers APC/C inactivation leading to synapse loss, neurodegeneration and memory and learning impairment (Gieffers et al., 1999; Almeida et al., 2005). Moreover, APC/C-Cdh1 activity is impaired in AD models and APC/C-Cdh1 targets accumulate in damaged areas of AD brains (Fuchsberger et al., 2017), which evidence an association between APC/C-Cdh1 activity and AD pathology. Then, the APC/C-Cdh1 signalling pathway may provide novel therapeutic strategies to modify AD progression.

## Structure and regulation of APC/C-Cdh1 in neurons

The APC/C complex is a cullin-RING E3 ubiquitin ligase that regulates cell cycle progression from M phase to the onset of S phase by targeting the degradation of cyclins and other regulatory proteins (Peters 2006; Watson et al., 2019). In vertebrates, the APC/C complex is composed by 19 subunits, distributed into three structural sub-complexes: the scaffolding platform, the tetratricopeptide repeat (TPR) arm and the catalytic core. The scaffolding platform connects the TPR arm to the catalytic core, which contains the catalytic subunits APC2, APC10, and APC11. The activation of APC/C requires the temporal and sequential binding of either Cdc20 or Cdh1, which are WD40-domain co-activator proteins that recruit APC/C substrates and increase APC/C specific activity by promoting its interaction with the E2 enzymes UbcH10 and UBE2S (Zhou et al., 2016; Watson et al., 2019). Despite these two activators, APC/C activity is also modulated by posttranslational modifications, including phosphorylation and acetylation, and inhibited by the mitotic checkpoint complex (MCC) and interphase early mitotic inhibitor 1 (Emi1) during cell cycle progression (Zhou et al., 2016). Briefly, cyclin-dependent kinases (Cdk) phosphorylate several APC/C subunits, hence promoting its association with Cdc20 in early mitosis. In contrast, Cdh1 phosphorylation by Cdks disrupts its interaction with APC/C. At late mitosis, Cdh1 dephosphorylation binds to and activates APC/C, ensuing Cdc20 inactivation through APC/C-dependent ubiquitination. Cdh1 maintains APC/C active from late anaphase through G1 phase (Zhou et al., 2016). Hyperacetylation of Cdc20 or Cdh1 inhibits APC/C activity (Kim et al., 2011).

The APC/C complex recognize its substrates through short destruction motifs or degrons, located at disordered regions of the substrate. The degrons are the D box (RxxLx[D/E][Ø]xN[N/S]) (Glotzer et al., 1991), the KEN box (residues KEN) (Pfleger and Kirschner 2000) and the ABBA motif (Fx[ILV][FY]x[DE]) (Di Fiore et al., 2015). Other non-canonical degrons are modifications of D boxes and KEN boxes. However, most proteins containing these degron consensus are unlikely to be targeted by APC/C because the degron must be available for recognition by the ligase (Davey and Morgan 2016).

**FIGURE 1:**
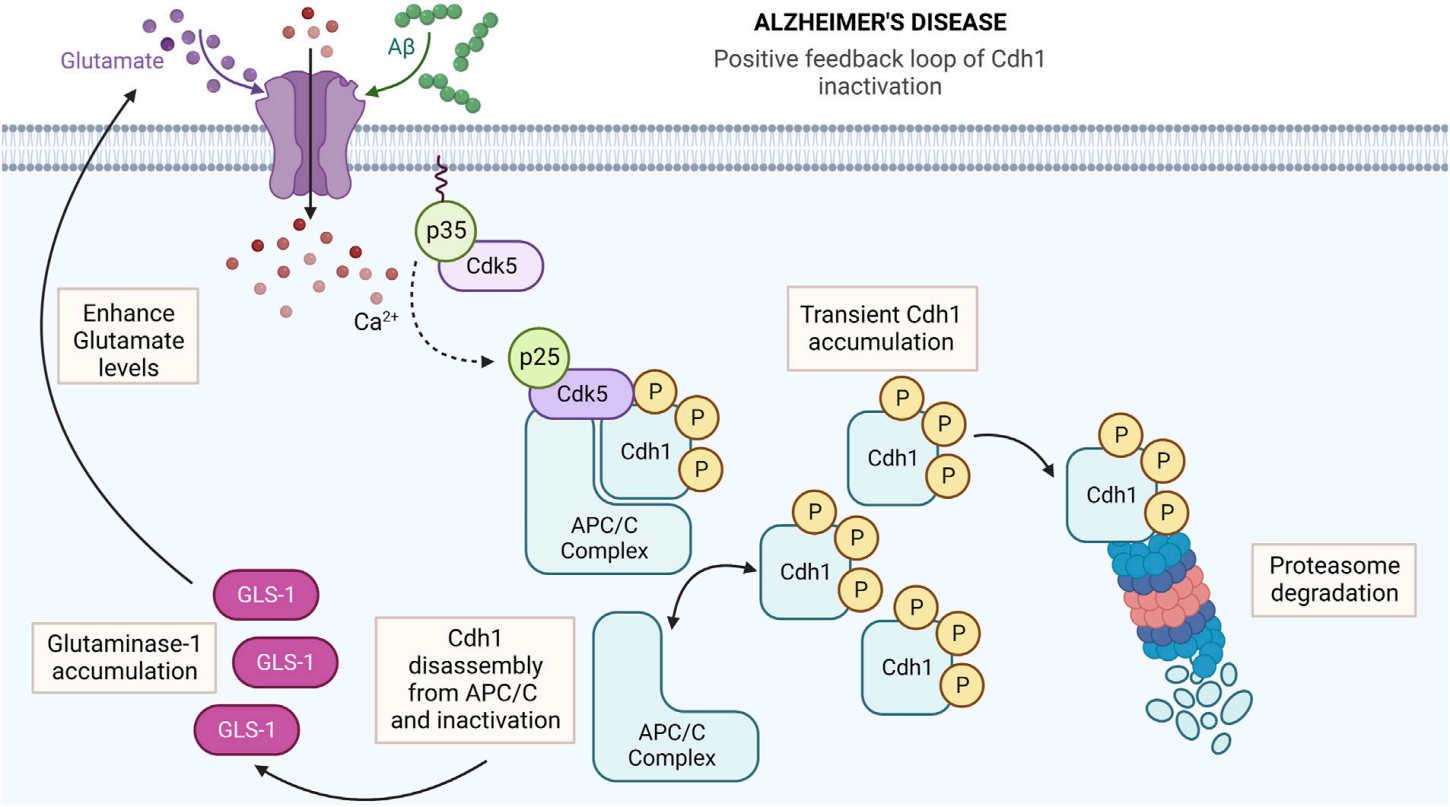
Amylod-β (Aβ) induces Cdk5-dependent Cdh1 phosphorylation, which triggers APC/C-Cdh1 inactivation. In AD pathology, Aβ induces p35 cleaved to p25 by the Ca^2+^-dependent calpain activation. The Cdk5-p25 binding causes Cdk5 over-activation, leading to Cdh1 phosphorylation, which causes APC/C-Cdh1 disassembly and inactivation, followed by a rapid Cdh1 accumulation. However, Cdh1 protein levels underwent a progressive decrease through time due to proteasome-dependent degradation of the protein. APC/C-Cdh1 inactivation also induces the glutaminase stabilization, which in turn enhance glutamate levels and intracellular Ca^2+^ influx, creating a positive feedback loop of Cdh1 inactivation that potentiates neurodegeneration. Created with BioRender.com.

In neurons, the main activator of APC/C is Cdh1 (Gieffers et al., 1999; Almeida et al., 2005; Bobo-Jimenez et al., 2017). APC/C-Cdh1 regulates crucial functions in the nervous system such as axonal growth (Konishi et al., 2004) synaptic size and plasticity (van Roessel et al., 2004; Fu et al., 2011; Huang et al., 2015; Bobo-Jimenez et al., 2017), neurogenesis (Delgado-Esteban et al., 2013), neuronal survival (Almeida et al., 2005; Bobo-Jimenez et al., 2017), and metabolism (Herrero-Mendez et al., 2009). APC/C-Cdh1 activity is tightly regulated in the brain by phosphorylation, subcellular localization, and protein stability. As occurs in proliferating cells (Zhou et al., 2016), dephosphorylated Cdh1 is mainly located in the nucleus where it binds APC catalytic subunits and activate APC/C (Maestre et al., 2008; Veas-Perez de Tudela et al., 2015b). In contrast, Cdh1 phosphorylation prevents its interaction with the complex and triggers Cdh1 translocation from the nucleus to the cytosol, all leading to APC/C-Cdh1 inactivation. We previously identified Cdk5 as the kinase responsible for Cdh1 phosphorylation in neurons (Maestre et al., 2008). Cdh1 protein stability is also autoregulated by the APC/C-proteasome pathway. Moreover, Cdh1 phosphorylation triggers a positive loop of APC/C inhibition leading to inactive Cdh1 stabilization in the cytosol (Veas-Perez de Tudela et al., 2015b) (Figure 1). Beside APC/C-Cdh1, the E3 ubiquitin ligase Skp1 Cullin1 F-box (SCF) also promotes Cdh1 instability in the nucleus (Listovsky et al., 2004; Nagai et al., 2018). Cdh1 degradation is promoted by forced localization of Cdh1 into the nucleus (Listovsky et al., 2004; Nagai et al., 2018). We previously identified a Asp187Gly mutation in Cdh1 that confines the protein into the nucleus, where it is ubiquitylated by SCF (Nagai et al., 2018), explaining the higher instability of mutated Cdh1 (Rodriguez et al., 2019).

## APC/C-Cdh1 function in neurodegeneration

Cdh1 is essential for neuronal plasticity and survival (Almeida 2012; Huang and Bonni 2016). In neurons, active APC/C-Cdh1 constantly prevents the accumulation of neurotoxic targets involved in cell cycle regulation, metabolism and redox homeostasis, and neuronal plasticity. Interestingly, dysregulation of these processes results in neurodegeneration, linking APC/C-Cdh1 targets to neurodegenerative diseases and particularly to AD (Dewanjee et al., 2022).

During brain development, Cdh1 regulates the differentiation of progenitor cells into neurons (Cuende et al., 2008; Delgado-Esteban et al., 2013), which switch to a postmitotic state. The activation of cell cycle machinery in neurons induces apoptosis rather than proliferation (Pandey and Vinod 2022). Moreover, the expression of cell cycle-related proteins, such as cyclin B1, has been detected in damaged areas of post-mortem AD brains (Vincent et al., 1997; Yang et al., 2003; McShea et al., 2007). Also, APC/C-Cdh1 activity downregulates cyclin B1 protein stability in neurons as an essential survival mechanism (Almeida et al., 2005; Almeida, 2012). Cdh1 loss induces cyclin B1 accumulation, which forces neurons to enter an aberrant cell cycle leading to neuronal apoptosis (Almeida et al., 2005). This effect is mimicked under excitotoxic conditions, which occurs in AD and stroke pathogenesis (Chamorro et al., 2016; Andersen et al., 2022). Thus, GluR stimulation induces Ca^2+^-dependent Cdk5 activation that phosphorylates Cdh1, which translocate from the nuclei to the cytosol. Consequently, APC/C is inactivated and cyclin B1 accumulated in both the nuclei (Maestre et al., 2008) and the mitochondria (Veas-Perez de Tudela et al., 2015a). Cdh1 phosphorylation and dissociation from APC/C also leads to the activation of cell cycle machinery by switching on a cyclin D1-Cdk4-pRb pathway leading to S-phase entry and neuronal apoptosis (Veas-Perez de Tudela et al., 2015b). In the mitochondria, cyclin B1 activates Cdk1 located in the inner mitochondrial membrane. The cyclin B1-Cdk1 complex phosphorylates the anti-apoptotic protein B cell lymphoma extra-large (Bcl-xL), causing ATP synthase inhibition, which induces oxidative damage, mitochondrial dysfunction, and neuronal apoptosis (Veas-Perez de Tudela et al., 2015a). Thus, cyclin B1 induces neuronal apoptosis by promoting cell cycle entry, mitochondrial dysfunction, and oxidative stress (Veas-Perez de Tudela et al., 2015a).

Impaired mitochondrial dysfunction and oxidative stress are characteristic features of AD involved in brain damage and cognitive decline (Dewanjee et al., 2022). Besides of cyclin B1, the accumulation of other APC/C-Cdh1 targets also promote mitochondrial damage and oxidative stress, which potentiate neurodegeneration. In this context, APC/C-Cdh1 targets the degradation of the regulatory glycolytic enzyme 6-phosphofructo-2-kinase/fructose-2, 6-bisphosphatase-3 (Pfkfb3), which is essential to actively downregulate glycolysis in neurons and maintain their redox homeostasis, all promoting neuronal survival (Almeida et al., 2004; Herrero-Mendez et al., 2009). In contrast, Cdh1 loss triggers Pfkfb3 stabilization, which provokes a metabolic switch by increasing glycolysis with a reduction in the pentose phosphate pathway, leading to oxidative damage, mitochondrial damage, and neurodegeneration (Herrero-Mendez et al., 2009). This effect occurs in parallel with cyclin B1-mediated apoptosis and knockdown of both cyclin B1 and Pfkfb3 fully abolished apoptosis in Cdh1 depleted cultured neurons. Thus, Cdh1 loss in neurons triggers apoptosis through both cyclin B1 and Pfkfb3 (Herrero-Mendez et al., 2009). Furthermore, GluRs activation stabilizes Pfkfb3, as well as cyclin B1 (Maestre et al., 2008), through the Cdk5-dependent phosphorylation and inactivation of Cdh1 (Rodriguez-Rodriguez et al., 2012), all leading to neuronal death. Interestingly, Pfkfb3 inhibition resulted in neuroprotection against an excitotoxic insult (Burmistrova et al., 2019), which poses Pfkfb3 to be an attractive target to fight against excitotoxicity.

APC/C-Cdh1 also regulates synaptic plasticity and learning and memory (van Roessel et al., 2004; Li et al., 2008; Fu et al., 2011; Poduri et al., 2012; Huang et al., 2015; Bobo-Jimenez et al., 2017). Loss of Cdh1 in neurons causes a decrease in LTP and impairs metabotropic-induced LTD in the hippocampus (Fu et al., 2011; Poduri et al., 2012; Huang et al., 2015). Moreover, APC2 depletion in excitatory neurons triggers APC/C-Cdh1 inactivation leading to cognitive impairment (Kuczera et al., 2011). Active APC/C-Cdh1 maintains dendritic network integrity and neuronal connectivity by degrading the dendrite destabilizer Rho protein kinase 2 (Rock2). Interestingly, Rock2 accumulates in the brain of AD patients (Herskowitz et al., 2013). Cdh1 knockout promoted dendrite disruption, synapse loss, and memory and learning impairment, leading to neurodegeneration. The administration of the Rock2 inhibitor, fasudil, prevented these pathological events (Bobo-Jimenez et al., 2017), confirming that the APC/CCdh1-Rock2 pathway regulates dendritic network integrity and brain functioning.

**FIGURE 2:**
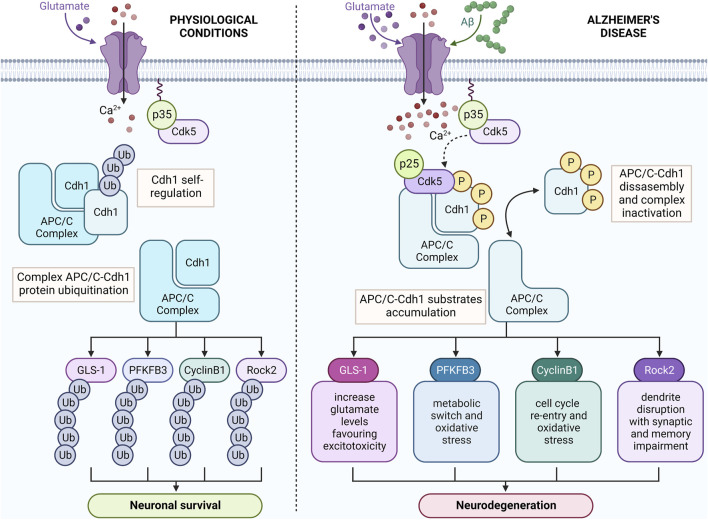
Amylod-β (Aβ) triggers APC/C-Cdh1 inactivation leading to the accumulation of its neurotoxic targets. Under physiological conditions, APC/C-Cdh1 constantly degrades the metabolic enzymes glutaminase 1 (GLS1) and 6-phosphofructo-2-kinase/fructose-2, 6-bisphosphatase-3 (Pfkfb3), the mitotic cyclin B, and the dendrite destabilizer Rho protein kinase 2 (Rock2) in neurons. In AD pathology, Aβ induces Cdk5-p25 hyperactivation that phosphorylates Cdh1 resulting in APC/C-Cdh1 inactivation and the accumulation of its targets, leading to neurodegeneration. Created with BioRender.com.

APC/C-Cdh1 constantly prevent neuronal cycle activation, mitochondrial dysfunction, oxidative stress, and synapse loss, all involved in the pathophysiology of neurodegenerative diseases, and particularly in AD (Dewanjee et al., 2022). This posits APC/C-Cdh1 targets in the pathogenesis of neurodegeneration. Under excitotoxic conditions, the accumulation of APC/C-Cdh1 targets would potentiate neurodegeneration by modulating different and complementary signalling pathways involving mitochondrial function (Figure 2). Furthermore, mitochondrial energy generation is necessary for neuroplasticity (Dewanjee et al., 2022). Therefore, APC/C-Cdh1 targets are interconnected and involved in neurodegeneration, then representing useful tools to develop new treatments for neurodegenerative diseases.

## APC/C-Cdh1 targets in Alzheimer’s disease pathology

Under physiological conditions, Cdk5 binds to its specific activators, p35 and p39, and regulates neuronal migration, neurite outgrowth, axonal guidance, and synaptic plasticity. Under stress, p35 and p39 are cleaved to p25 and p29, respectively, by Ca^2+^-dependent calpain activation. The Cdk5-p25 complex is stable and cause Cdk5 over-activation, thereby hyperphosphorylating several substrates, including Cdh1 (Maestre et al., 2008; Veas-Perez de Tudela et al., 2015a), leading to neurodegeneration (Ao et al., 2022). This hyperactive Cdk5-p25 complex contributes to AD pathogenesis (Ao et al., 2022; Maitra and Vincent 2022). Furthermore, oligomerized Aβ stimulates intracellular Ca^2+^ influx *via* the N-methyl-d-aspartate (NMDA)-type GluR, leading to Cdk5-p25 activation and Cdh1 phosphorylation (Lapresa et al., 2019; Maitra and Vincent 2022), then linking Cdh1 inactivation to AD pathology (Lapresa et al., 2019) (Figure 1). Considering that there is no drug aimed to maintain or enhance Cdh1 activity, acting downstream in the Cdh1 pathway opens new possibilities for drug development. Thus, APC/C-Cdh1 targets arises as promising therapeutic targets to fight against AD progression.

The Aβ-induced Cdk5 activation phosphorylates Cdh1 and cause the early APC/C-Cdh1 inactivation, evidenced by Cdh1-APC3 disassembly followed by Cdh1 accumulation (Lapresa et al., 2022). Nevertheless, Cdh1 protein levels underwent a progressive decrease through time (Lapresa et al., 2022) due to proteasome-dependent degradation of the protein (Fuchsberger et al., 2016). APC/C-Cdh1 inactivation induces the accumulation of its target, glutaminase (Colombo et al., 2010), which in turn enhance glutamate levels and intracellular Ca^2+^ influx, creating a positive feedback loop of Cdh1 inactivation in AD (Fuchsberger et al., 2016) (Figure 1). Therefore, the activity of APC/C-Cdh1 must be carefully regulated to evade any cellular imbalance that culminate in neurodegeneration.

In experimental models of AD, glutaminase becomes accumulated due to the degradation of Cdh1, which, in turn, increases glutamate levels enhancing neurotoxicity (Fuchsberger et al., 2016). Accordingly, high levels of glutaminase and glutamate are found in the brains of AD patients (Burbaeva et al., 2005; Madeira et al., 2018). In this context, memantine, an NMDA receptor antagonist, has been tested in clinical trials of AD. Although the patients showed an improvement in cognition and behaviour, the benefits on the trial outcomes were modest (Kishi et al., 2017). However, glutaminase inhibition prevents excitotoxicity and Aβ-induced apoptosis *in vitro* (Fuchsberger et al., 2016). Recently, the glutaminase antagonist JHU-083 was shown to normalize hippocampus glutaminase activity in an AD mouse model, eliciting an improved cognition response (Hollinger et al., 2020). However, the toxic side effects and high clearance of JHU-083 (Yang et al., 2021) preclude its chronic use for AD treatment, indicating the need to continue the research of other glutaminase inhibitors as potential therapeutic targets in AD.

Another metabolic enzyme target of APC/C-Cdh1 is the glycolytic regulator Pfkfb3 (Herrero-Mendez et al., 2009). Under an excitotoxic stimuli, including Aβ-induced GluR activation, Pfkfb3 becomes stabilized (Rodriguez-Rodriguez et al., 2012). The Cdk5-APC/C signalling pathway promotes Pfkfb3 accumulation and its translocation from the nucleus to the cytosol, which switches neuronal metabolism leading to oxidative stress and neuronal death (Rodriguez-Rodriguez et al., 2012). Recently, Traxler et al. (2022) described a metabolic switch to glycolysis in neurons from AD-patient-derived fibroblasts, which enhances neuronal vulnerability to apoptosis. Pharmacological inhibition of Pfkfb3 with the small molecule inhibitor AZ67 prevented Aβ-induced neuronal damage (Burmistrova et al., 2019). Thereby, drugs targeting glycolytic enzymes, such as the APC/C-Cdh1 substrate Pfkfb3, might provide new disease-modifying therapeutic strategies for this neurological disorder.

The adult brain contains two main neurogenic niches, the ventricular-subventricular zone and the granular layer of the dentate gyrus in the hippocampus. Although direct evidence of human adult neurogenesis remains elusive, indirect approaches point to the existence of adult neurogenesis in the hippocampus of healthy humans throughout their lives (Moreno-Jimenez et al., 2021). Moreover, immature neurons have an essential role in hippocampus-dependent learning (Sahay et al., 2011). Impaired neurogenesis has been detected at early stages in AD, which would promote cognitive decline of AD patients (Moreno-Jimenez et al., 2019). Furthermore, hippocampal neurogenesis inhibition triggers neuronal death and cognitive decline progression in transgenic AD mouse models (Culig et al., 2022). Interestingly, Cdh1 induces neuronal progenitors exit of the cell cycle and the onset of differentiation during brain development (Delgado-Esteban et al., 2013). Cdh1 loss shortens G1 phase and enhances S phase, leading to replicative stress and p53-mediated apoptosis of neural progenitor cells (Delgado-Esteban et al., 2013). Recently, Esteve et al. (2022) found increased Cdh1 levels, decreased cyclin B1 levels, and reduced proliferation of neuronal progenitors in the ventricular-subventricular zone of AD mice, which triggers senescence. Adult neurogenesis stimulation by the regulation of Cdh1 and/or cyclin B1 levels might be important to modify AD progression. However, and in contrast to progenitor cells, cyclin B1 accumulates in postmitotic neurons in AD (Vincent et al., 1997; Yang et al., 2003; McShea et al., 2007), leading to neurodegeneration (Maestre et al., 2008; Veas-Perez de Tudela et al., 2015a). Therefore, cell-type specific strategies should be considered to establish AD therapies based on Cdh1 targets modulation.

APC/C-Cdh1 also regulates levels of the dendrite destabiliser Rock2 (Bobo-Jimenez et al., 2017), which accumulates in the AD brain and protein levels remains elevated throughout disease progression (Herskowitz et al., 2013). Recently, Lapresa et al. (2022) described that Aβ-induced Cdh1 phosphorylation by Cdk5 induces Rock2 stabilization in neurons, leading to neurodegeneration and memory loss. Furthermore, Rock2 inhibition decreases amyloid-β levels (Herskowitz et al., 2013) and prevents neuronal death caused by oligomerized Aβ (Song et al., 2013; Lapresa et al., 2022). The Rock inhibitor fasudil has been approved for clinical use. Despite being effective (Bobo-Jimenez et al., 2017), fasudil does not specifically inhibit Rock2 and therefore other specific compounds have been developed. In this context, the specific Rock2 inhibitor SR3677 attenuates neuronal apoptosis and memory impairment caused by Aβ oligomers in mice (Lapresa et al., 2022). Therefore, targeting Rock2 is a promising strategy to effectively tackle AD progression.

## Conclusion

Over the past decade, huge efforts have been made to find suitable targets for the treatment of AD. Most of the attempts have tried to tackle Aβ. However, none of the Aβ-based therapies have been effective so far, with the controversial exception of the FDA approved aducanumab (Cummings et al., 2022). A refocusing approach should be made addressing new targets further downstream of Aβ accumulation in the AD pathology.

Through Cdh1 activation, the APC/C complex maintains synaptic plasticity and neuronal survival, which is essential for brain functioning and healthy aging. APC/C inactivation has been found in AD models, Cdh1 targets being accumulated in the brain of AD patients, which promote neurodegeneration and cognitive decline through several mechanisms including cell cycle machinery activation, excitotoxicity, metabolism impairment, oxidative stress, and synaptic dysfunction (Figure 2), rendering this pathway as a suitable candidate to develop drugs for AD treatment. Understanding the APC/C-Cdh1 signalling pathway in AD might be important to identify new molecular targets for the development of effective disease-modifying treatments for this neurological disorder.
